# Evaluation of Face Validity and Acceptability of the Care Partner Hospital Assessment Tool

**DOI:** 10.1093/geroni/igad011

**Published:** 2023-02-06

**Authors:** Beth Fields, Madelyn Carbery, Richard Schulz, Juleen Rodakowski, Lauren Terhorst, Catherine Still

**Affiliations:** Department of Kinesiology, University of Wisconsin-Madison, Madison, Wisconsin, USA; Department of Kinesiology, University of Wisconsin-Madison, Madison, Wisconsin, USA; Shirley Ryan Ability Lab, Chicago, IL, USA; University Center for Social and Urban Research, University of Pittsburgh, Pittsburgh, Pennsylvania, USA; Department of Psychology, University of Pittsburgh, Pittsburgh, Pennsylvania, USA; Department of Occupational Therapy, University of Pittsburgh, Pittsburgh, Pennsylvania, USA; Department of Occupational Therapy, University of Pittsburgh, Pittsburgh, Pennsylvania, USA; Department of Kinesiology, University of Wisconsin-Madison, Madison, Wisconsin, USA

**Keywords:** Care partners, Hospital, Implementation, Instrumentation study, Older adults, Screening tool

## Abstract

**Background and Objectives:**

Care partners of hospitalized older adults report their caregiving needs are not being addressed. The Care Partner Hospital Assessment Tool (CHAT) is a feasible and appropriate tool for practitioners’ use with care partners in the hospital setting. This article explores the face validity and acceptability of the CHAT among care partners of hospitalized older adults.

**Research Design and Methods:**

A qualitative descriptive study was used to identify common themes among care partners’ responses from semistructured interviews. The CHAT was administered to care partners of older adults admitted to a medical–surgical unit in an academic medical center in Madison, WI, from October 2021 to January 2022. A semistructured, follow-up interview was completed by the same care partners after discharge. Interviews were transcribed and coded for themes to capture overall impressions of the CHAT. Care partners addressed the usefulness, comfort, content, and complexity of the CHAT.

**Results:**

Twelve care partners participated in the study. Care partners reported that the CHAT was easy to understand and complete, was judged to be useful to both the care partner and older adult, and helped identify care partner needs. Care partners suggested ways to improve the tool including administration, additional content areas to include, and modes of delivery.

**Discussion and Implications:**

The results establish the face validity of the CHAT and support the acceptability of the tool for use with care partners of hospitalized older adults.

**Translational Significance:** Care partners of hospitalized older adults frequently report their needs are not being identified by healthcare practitioners and are not receiving the necessary training to complete their caregiving responsibilities. The Care Partner Hospital Assessment Tool (CHAT) was developed to facilitate practitioners’ timely identification and training of care partners. Findings from this study demonstrate that the CHAT has strong face validity and is acceptable for identifying and addressing care partners’ caregiving needs.

The U.S. healthcare system relies heavily on unpaid care partners (friends and family) of older adults (65+ years) to provide care after hospitalization. These care partners provide an estimated $470 billion in care labor totaling about 34 billion hours of care, which drastically reduces the number of resources expended by our hospital systems (Reinhard, Feinberg, et al., 2019). Despite our healthcare system’s dependence on unpaid care labor, care partners of older adults report their needs are not being identified by healthcare practitioners and are not receiving the necessary training to complete their caregiving responsibilities (Hunt & Reinhard, 2015). This is problematic because when care partners’ needs go unidentified and unaddressed, they are at a high risk of experiencing excess burden, chronic stress, and depression, which, in turn, can negatively affect the older adult care recipients (Bauer et al., 2011; Schultz & Eden, 2016; Shyu, 2000). Additionally, care partners “consistently rate their level of engagement in decision-making about discharge plans and the quality of their preparation for the next stage of care as poor” (Naylor & Keating, 2008, p. 2).

Although research demonstrates that systematically including care partners in hospital care improves health outcomes and reduces post-discharge costs and resource use (Rodakowski et al., 2017), an American Association of Retired Persons report challenges the U.S. healthcare system to restructure and effectively identify and train care partners in daily practice (Reinhard, Young, et al., 2019). Poor care partner and healthcare system outcomes may be attributed to the lack of screens, assessment, or clinical decision-support tools available to assist healthcare practitioners in identifying and addressing care partners’ preferences and caregiving needs (Fields et al., 2018; National Alliance for Caregiving, 2021).

A recent report from the National Alliance for Caregiving emphasized clinical decision-making tools are essential to assisting practitioners in incorporating care partners into healthcare practice (National Alliance for Caregiving, 2021). The RAISE Family Caregivers Act (Public Law 115–119), signed into law in 2018, mandated the development of a national family caregiving strategy to help systemically identify and address care partners’ preferences and training needs (RAISE Family Caregiving Advisory Council, 2021). The 2022 National Strategy to Support Family Caregivers describes concrete actions that federal, state, and local communities and agencies can take to achieve desired outcomes (The Recognize, Assist, Include, Support, and Engage [RAISE] Ace Family Caregiving Advisory Council & The Advisory Council to Support Grandparents Raising Grandchildren, 2022). For example, one action is focused on better equipping healthcare practitioners with screening and decision-support tools to gather accurate information about the caregiving context. Clinical decision-support tools are important because they can aid in providing timely information and suggestions for care delivery processes to interprofessional teams, thereby improving efficiency and potentially lowering costs (Berner, 2009). A critical need exists for valid decision-making tools to facilitate practitioners’ timely identification and training of care partners.

In response to this need, the Care Partner Hospital Assessment Tool (CHAT) was developed to facilitate practitioners’ timely identification and training of care partners of cognitively unimpaired older adult patients during their hospitalization. Guided by the widely used and effective decision-support model of Screening, Brief Intervention, and Referral to Treatment (Agerwala & McCance-Katz, 2012), the CHAT applies a sequential screening and referral pathway to identify and train care partners. In particular, the CHAT prompts care partners to answer questions about their background, preferences for care during and after the patient’s hospitalization, and information or training needs they may have to fulfill caregiving responsibilities once the patient is discharged from the hospital. After care partners complete the CHAT, their responses are reviewed by the patient’s medical team to determine what services, if any, they should be referred to during the patient’s hospital stay (see Supplementary Material Section 1 for a copy of the CHAT).

The CHAT has strong content validity and is endorsed as appropriate and feasible by practitioners in the hospital setting (Carbery et al., 2021; Fields et al., 2021). As a next step toward advancing the measurement validity and implementation of the CHAT, the face validity and acceptability of the tool to care partners are needed. Face validity implies that the tool appears to capture what it is intended to, and acceptability describes the usefulness, complexity, comfort, and content of the tool (Connell et al., 2018; Portney & Watkins, 2009; Proctor et al., 2011). It is critical to understand these measurement validity and implementation outcomes from the care partner perspective before clinical utility to facilitate the adoption of the CHAT into an organization or system (Proctor et al., 2011). Indeed, evidence demonstrates that research involving stakeholders’ real-world needs and concerns contributes to improved outcomes such as efficiency and quality of health care service delivery (Bombard et al., 2018; Heckert et al., 2020).

Therefore, the purpose of this qualitative descriptive study was to establish the face validity and acceptability of the CHAT among care partners of hospitalized older adults. We asked two primary research questions: Does the CHAT appear to identify and address care partners’ caregiving preferences and needs? Does the CHAT make sense and is the tool easy to use? Information gleaned from this study can help address fundamental gaps in hospital care processes by identifying and addressing care partners’ preferences and caregiving needs.

## Method

### Study Design and Recruitment

**Figure 1.**
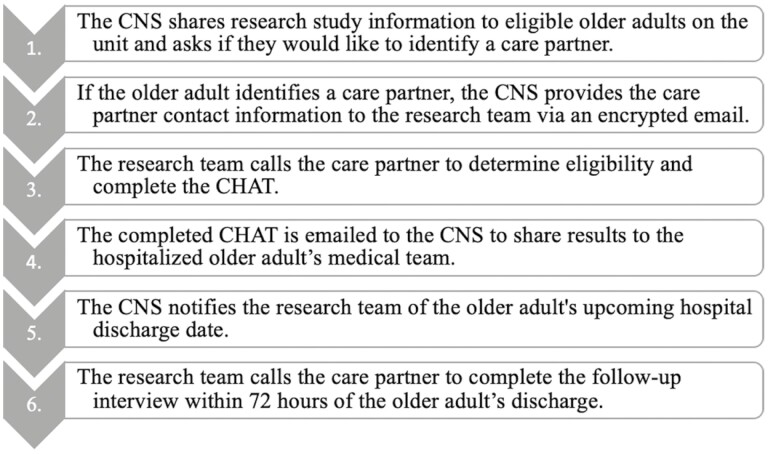
Workflow for the CHAT administration and distribution of results. CNS = Clinical Nurse Specialist; CHAT = Care Partner Hospital Assessment Tool.

To examine the face validity and acceptability of the CHAT, we conducted a qualitative descriptive study with care partners of hospitalized older adults on a medical–surgical unit at the University of Wisconsin (UW) Health, a large, academic medical center in Madison, WI (Sandelowski, 1991). Prior to participant recruitment, the research team completed two virtual trainings (due to coronavirus disease [COVID-19] visitor restrictions put in place) with a Clinical Nurse Specialist (CNS) for Research and Evidence Based Practice at the UW Health to determine workflow for the administration and distribution of CHAT results to the hospitalized older adult’s medical team for research purposes only (see Figure 1). Previous CHAT research describes one example of how to implement the tool into clinical workflow (Carbery et al., 2021). We worked with the CNS to conveniently recruit care partners with the following eligibility criteria: (a) provide unpaid care either before, during, and/or after the hospitalization of an older adult (65+ years); (b) speak and/or understand English; and (c) be at least 18 years of age. Care partners were contacted for eligibility screening after being identified as a primary care partner by an older adult that was admitted to the hospital. Older adult patients admitted as observation were excluded from this study given the challenges associated with visitor restrictions and the ability to contact care partners in a timely manner. This study was reviewed and approved by the UW-Madison Institutional Review Board (protocol ID: 2019-1089) as exempt. All recruited care partners provided verbal consent to participate in the study.

### Data Collection

Within 24 hours of an older adult’s hospital admission, their identified care partners participated in a 15- to 30-minute intake interview over the phone (due to COVID-19 visitor restrictions put in place) with a trained member of our research team. The purpose of this interview was to share information about the research study and obtain verbal consent for participation, gather demographic information, and complete the CHAT with the care partner. The research team then shared the completed CHAT with the CNS, who then forwarded the care partners’ responses to the hospitalized older adult’s medical team. Within 72 hours of the older adult’s hospital discharge, care partners participated in another 15- to 30-minute follow-up interview over the phone (due to COVID-19 visitor restrictions put in place) with a trained member of our research team. The purpose of this interview was to gain a deeper understanding of their opinions, suggestions, and experience of completing the CHAT (see Supplementary Material Section 2 for a complete interview guide). We chose to complete the interviews within 24 hours of admission and 72 hours from discharge to quickly capture whether the care partner received any information or skills training during hospitalization and enhance care partner recall of hospital experience. All interviews were audio recorded, transcribed verbatim, and deidentified. Because of COVID-19 and restrictions for collecting data in-person at the hospital, participants were mailed a $50 gift card for completing both interviews.

### Data Analysis

Data were uploaded, stored, and managed in NVivo 12 Pro (QSR International Pty Ltd., n.d.). A trained member of the research team completed a thematic analysis using a three-step inductive approach, which included (1) a familiarization phase (getting to know the data by rereading transcripts and writing memos), (2) an organization phase (coding/searching for themes, categorizing, and reviewing themes), and (3) a reporting phase (selection of themes that directly answer the research question; Sandelowski, 1991; Vaismoradi et al., 2013). In the familiarization phase, the research team members read through transcripts, created memos, and decided on an inductive approach to thematic analysis. In the organization phase, the same research team member developed an open coding system to track the frequency of occurrences of specific themes. The coding scheme was then triangulated with two other qualitatively trained research team members to increase the reliability and trustworthiness of the generated results. In the reporting phase, all research team members came together for a hybrid meeting to compare coding and discuss disagreements and interpretations of data until a group consensus was reached.

## Results

Sixteen care partners were contacted via referral from hospitalized older adults and 12 care partners participated in the study. Data saturation was reached after 12 care partners so recruitment was ceased (Streiner et al., 2015). Seventy-five percent of care partners lived with and identified as being the spouse of the hospitalized older adults. The other 25% of care partners lived separately from and identified as being adult children of the hospitalized older adults. Additional demographic information is listed in Table 1.

**Table 1.** Demographic Characteristics of Care Partners (*N* = 12)

Demographic characteristic	Frequency (%)
Female	8 (67)
Healthcare background	
Administration	2
Education	1
Age	
40–50	3 (25)
50–60	3 (25)
60–70	3 (25)
70–80	2 (17)
80+	1 (8)
Highest level of education	
High school	5 (42)
Some college	2 (17)
College or beyond	5 (42)
Working status	
Not working/unemployed	2 (17)
Part time	1 (8)
Full time	5 (42)
Retired	4 (33)

Although each caregiving situation was unique to the hospitalized older adult, care partners were similar in their overall views of the CHAT. The following themes were revealed: purpose, usefulness, content, comfort, and complexity. Additional quotes supporting each theme are reported in Table 2.

**Table 2.** Additional Quotes From Care Partners about the CHAT

Theme	Participants comments
Purpose	• I got relief to know that when you reached out and offered this, I’m like, Oh my gosh, somebody is there to help me (P4). • It’s wonderful that you consider the needs of the caretaker while the patient is hospitalized and help address their needs from the get-go. This is very smart (P1). • I think it is valuable to go through and ask this stuff (P9).
Usefulness	• Getting help and training like this would be extremely useful (P8). • I would definitely be able to benefit from some of the things she mentioned (in the CHAT) (P6). • If I had filled out the CHAT earlier, it potentially could have been a preventative from some of negative experiences we went through (P6). • I think it would improve communication with health care practitioners … improve the amount of information we are given and the quality of information we [care partners] are given (P7). • It would better prepare me for whatever is going on, whether it’s his [patient] hospitalization or in home care (P2).
Content	• Some of the questions on the CHAT weren’t relevant to me, but that doesn’t mean they aren’t relevant to someone else (P9). • You covered a lot of useful information (P12). • The CHAT covers everything pretty well (P8). • The CHAT is very thorough (P9). • It’s a nice little package of questions (P5).
Comfort	• I thought it [the CHAT] was pretty straightforward and intuitive (P2). • They explained it [the CHAT] to me pretty well (P10). • I absolutely 100% thought the process was straightforward (P8). • I like how the CHAT is broken down into sections so it gave myself an indication as to how much more time you’re going to need or try to gather my thoughts in preparation of what will be asked next (P4). • I don’t think it [the CHAT] was very long at all. Not too long or too short (P7). • I thought it [the CHAT] went pretty quick (P8).
Complexity	• This tool could help me kind of understand his [family member] needs and precautions in the community (P3). • I like the idea of maybe a QR code or electronic version (P4). • If they [care partners] are getting resistance from their spouse, a kiosk would allow them to maybe answer those questions freely right within that space (P3). • Over the phone was fine with me (P11).

*Note*: CHAT = Care Partner Hospital Assessment Tool.

### Purpose

Care partners agreed that the CHAT was useful for identifying and addressing their needs. Care partners reported they had not seen or been offered anything like the CHAT over the course of other healthcare experiences. One care partner shared the CHAT would better prepare him for his caregiving responsibilities during and post-hospitalization (P9). Participant 3 added, “If you could get this going in healthcare systems, you [care partners] wouldn’t feel so alone” (P3). Another care partner mentioned how the CHAT would have been beneficial during an emergency room visit in addition to using a medical–surgical unit (P6).

### Usefulness

#### Impact on care partner

Care partners detailed various benefits of the CHAT on their caregiving responsibilities and personal well-being. For example, one care partner emphasized how the CHAT made her feel more confident in her caregiving and another appreciated the acknowledgment of her skills training needs even as a veteran (or more experienced) care partner (P7 and P8). Specifically, stating, “It [the CHAT] made me feel more confident about what I’m doing, and it just makes me feel more confident that I can go forward doing the right thing” (P7). Additionally, care partners reported the CHAT could increase communication with the medical team and prompt discussions and/or concerns that may not have been considered without completion of the CHAT.

#### Impact on older adult

Care partners acknowledged that if they received more support, education, or skills training due to the completion of the CHAT, the older adult may receive better care after returning home post-discharge. One care partner admitted that he wished he had completed the tool years ago to better understand his wife’s condition and know how to actively participate in her care (P10).

### Content

Many care partners shared that they liked the diverse content of the CHAT and thought that the tool addressed their caregiving responsibilities, stating “I liked how you guys categorize the different topics to cover the broad spectrum (of caregiving topics)” (P11). Care partners shared suggestions about additional content for caregiving questions. For example, care partners mentioned adding questions addressing the next steps in care (home health, specialty referrals, follow-up appointments), support groups of care partners, and reviewing test/lab results with the hospitalized older adult’s medical team. Although the majority of care partners reported that the vocabulary used on the CHAT was easy to understand, one participant suggested replacing the word “tool” with “questionnaire” to avoid confusion (P1).

### Comfort

#### Format of the CHAT

All the care partners reported the CHAT was an acceptable length for the information being gathered. One care partner shared it felt doable in comparison to other questionnaires and appreciated the clarity of instructions and breakdown of sections (P4). She added, “I thought it was a good length. I like that you recap it in the beginning and how you broke it down into sections which gave myself an indication as to how much more time you’re going to need” (P4). Another shared the experience of completing the CHAT felt “straightforward” (P2).

### Complexity

#### Delivery of the CHAT and follow-up training

Care partners had varying opinions about the administration of CHAT and received follow-up support to address their caregiving preferences and needs. Some care partners preferred to complete the CHAT over the phone for the convenience and accessibility during COVID-19 visitor restrictions and reluctance to travel to a hospital. In contrast, some care partners preferred to complete the CHAT in-person alongside a hospital staff member and/or their family members in case questions arose. There were mixed reviews on whether the CHAT should be completed digitally or with pen and paper. Multiple care partners reported difficulty remembering the CHAT’s purpose or how they had answered the questions on the CHAT without a hard copy to reference. For example, a care partner stated, “I have to be perfectly honest with you, it’s all a blur what we talked about last week, I’ve been so stressed lately” (P9).

Care partners reported a desire to receive training in-person for nursing- and medical-related tasks and printouts or conversations for other caregiving responsibilities. Multiple care partners added having a digital or hard copy of the CHAT to refer to during discussions with the older adult’s medical team or future hospitalizations would be helpful.

#### Visitor restrictions due to COVID-10 pandemic

Care partners overwhelmingly reported visitor restrictions due to the COVID-19 pandemic as a barrier to completing the CHAT effectively and receiving the necessary training. Care partners felt they would have understood their caregiving needs better if they were able to visit their family members in the hospital and, in turn, be able to communicate more effectively with various healthcare practitioners. For example, a care partner described constantly missing medical rounds because she was limited in the time frames she was able to visit her husband due to the COVID-19 restrictions (P3). Another expressed, “Quite honestly, if the CHAT was administered in person, it probably wouldn’t have happened. I did not like walking your [hospital’s] hallways with COVID-19, so in-person, I wouldn’t have been happy [to participate in study]” (P9).

#### Unknown discharge status

Care partners described difficulty envisioning their family members’ discharge needs, and, in turn, reported a challenge in knowing what their caregiving responsibilities might entail. For example, one care partner didn’t know what training she would need because she was unsure of her family members’ hospital length of stay and functional deficits at the time of completing the CHAT (P2).

## Discussion

Our study established face validity for the CHAT by synthesizing care partner qualitative feedback. Care partners expressed that the CHAT was relevant for capturing care partners’ personal information, plans and preferences, and skills and supports. Feedback also revealed that the CHAT was appropriate for care partners with respect to vocabulary, length, and clarity of instructions. Overall, care partners voiced their satisfaction with using the CHAT to identify and address their needs. The research team considered the inclusion of care partner ideas to construct additional items and improve future implementation of the CHAT.

CHAT has the potential to increase care partners’ confidence by better preparing care partners for their responsibilities after discharge. Care partners who feel confident in assuming their care responsibilities may lead to fewer accidents, medical errors, or readmissions (Hartke et al., 2006; Hunt & Reinhard, 2015). Additionally, high care partner burden, frequently associated with unpreparedness, can lead to poor health outcomes including physical injury, depression, and emotional and financial strain, so the CHAT could reduce these risks (Hunt & Reinhard, 2015; Kaiser & Kaiser, 2017). The older adult care recipient may also benefit from a reduced caregiver burden. For example, one study found that reduced caregiver burden may decrease mortality risk in older adults (Pristavec & Luth, 2020). Furthermore, the diversity of CHAT’s content ensures that all care partners’ various caregiving responsibilities are addressed. As our population continues to age, care partners are taking on more complex responsibilities, including medical care, home-making tasks, personal financing, transportation, care coordination, social/emotional support, and activities of daily living (dressing, toileting, bathing, etc.; Hunt & Reinhard, 2015; Reinhard, Young, et al., 2019; Schultz & Eden, 2016). The CHAT’s content reflects the progressive demands of care partners and supports the mission of the RAISE Family Caregivers Act (RAISE Family Caregiving Advisory Council, 2021). Furthermore, the expansive topics covered in the CHAT may make the tool useful in settings other than medical–-surgical units, such as the emergency room or primary care.

The best clinical practice supports effective communication between healthcare practitioners, patients, and their care partners (Hunt & Reinhard, 2015; The Joint Commission, 2011). The Joint Commission, an accreditation organization for hospital systems, provides a roadmap for increasing communication and family-centered care that includes (a) identifying care partners early; (b) communicating information between patients, families, and medical teams; and (c) engaging families in discharge processes (The Joint Commission, 2010). Clinical decision-support tools, like the CHAT, can increase interprofessional communication and provide practitioners a tool to meet the Joint Commission and RAISE Act standards (RAISE Family Caregiving Advisory Council, 2021; The Joint Commission, 2010). Care partners felt like the CHAT could improve communication between families and healthcare practitioners and appreciated the opportunity to discuss concerns related to their information and skills training needs. Feeling valued and heard by healthcare practitioners via the CHAT came without participant burden. For example, care partners emphasized that the CHAT was an appropriate length, and found the instructions to be clear, so its communication benefits outweigh its deterrents to completion.

Before the clinical outcomes of the CHAT can be examined, we must understand the considerations for implementing CHAT effectively into a system or organization (Proctor et al., 2011). Individuals are more likely to complete clinical decision-making tools, like the CHAT, when the tool is meaningful, relevant, and convenient to complete (McCambridge et al., 2011; Singer & Ye, 2013). Care partners emphasized the CHAT was pertinent to their caregiving roles and responsibilities; however, there were multiple suggestions offered on how to deliver the CHAT to increase convenience. For example, the CHAT may be successful in both written and digital forms, which were both modes of delivery suggested by care partners. There are limited differences between written and digital response rates, and offering both styles of delivery is especially beneficial for the older adult population (Hohwü et al., 2013; Kelfve et al., 2020). The flexibility of offering multiple modes of delivery may make the CHAT a versatile, adaptable tool.

An added layer of complexity for implementing the CHAT is the language used to describe the tool and its purpose. Older care partners (aged 70+) verbalized confusion with the CHAT due to difficulty recalling its purpose. Completing the assessment over the phone may have added to difficulty remembering the CHAT’s specific questions and how individual care partners responded. Offering a hard copy of the completed CHAT in addition to discussing the tool may clarify the CHAT’s purpose and enhance its usability, especially for older care partners (Kelfve et al., 2020).

Another intricacy of implementing the CHAT is determining when to deliver the tool during an older adult’s hospitalization. Care partners described not knowing the discharge condition or status of the hospitalized older adult upon admission, which proved to be a challenge to completing the CHAT. This uncertainty made it difficult for care partners to know whether he/she needed education or skills training on a caregiving topic based on its relevance to the older adult. A proposed solution to this problem would be to administer the CHAT at multiple time points across hospitalization or readminister it across the continuum of care based on changes in the older adult’s health status. Many assessments used within the hospital setting are administered multiple times to track change or prove the attainment of skills, such as pain assessments (Gordon et al., 2008; New York State Department of Health, 2022; The Joint Commission, 2022).

### Strengths and Limitations

There were several strengths of this study. The research team met multiple times during the data analysis phase to triangulate interview codes to increase validity and gain a thorough understanding of the data. The study recruited end-user stakeholders who may benefit from CHAT’s future implementation and utilization. Finally, the study built on past research related to the CHAT to deepen and broaden our understanding of how the tool may be used within a large academic medical center.

There were several limitations of this study. Common demographic and contextual characteristics are not collected as part of the CHAT, including race, ethnicity, language, and caregiving demands. Although these characteristics can affect care partner perspectives and predisposition to complete the tool, previous research revealed the need to reduce the original number of items on the CHAT given the fast-paced nature of hospital care (Carbery et al., 2021). Future research should explore whether the CHAT should be adapted to meet specific caregiving and cultural contexts (e.g., dementia caregiving in Hispanic communities). The varied gap between the administration of the CHAT and the follow-up interview due to older adults’ length of stay and COVID-19 visitor restrictions may have biased care partner recall or their experience with the CHAT. Future research should evaluate the influence of length of patient stay in hospital on care partners’ experiences with the CHAT. Finally, there is limited transferability and generalizability due to the study occurring in one medical–surgical unit at a large, academic medical center. Findings may be influenced by social determinants of the region. Future research to examine the CHAT’s clinical outcomes like team communication could provide insight into the synergies among care partners, older adults, and healthcare practitioners.

## Conclusion

The CHAT has strong face validity and is acceptable for identifying and training care partners of hospitalized older adults. The purpose and content of the tool have the potential to be useful and beneficial to not only care partners, but also older adults and healthcare practitioners. The CHAT may help fill the gap for an evidence-supported, clinical decision-making tool targeted at systematically integrating care partners into hospital care processes.

## Supplementary Material

igad011_suppl_Supplementary_Material
