# Supplementary material for: Evaluation of Face Validity and Acceptability of the Care Partner Hospital Assessment Tool
Source: Innov Aging. 2023 Feb 6;7(2):igad011. doi: 10.1093/geroni/igad011 (PMC10078971; doi:10.1093/geroni/igad011)
Supplement: igad011_suppl_Supplementary_Material [file igad011_suppl_supplementary_material.docx]

**Section 1: Care Partner Hospital Assessment Tool (CHAT)**

**
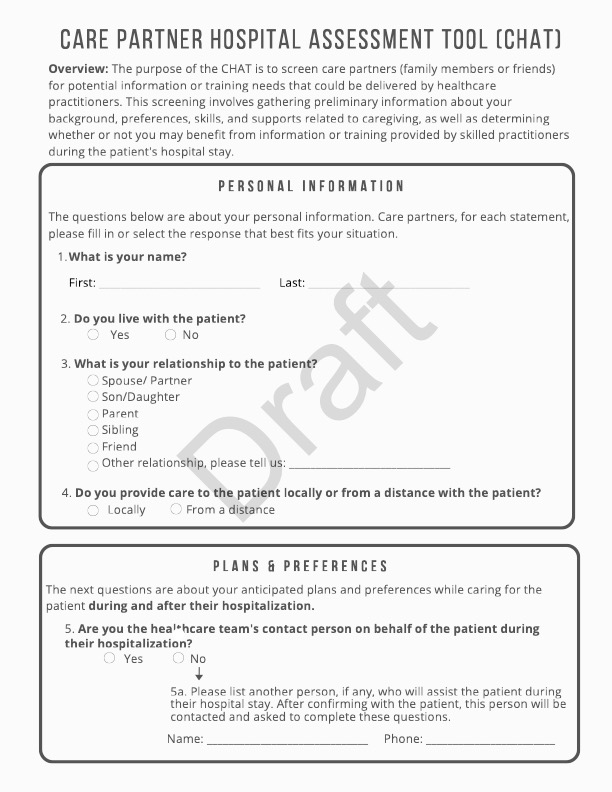
**

**
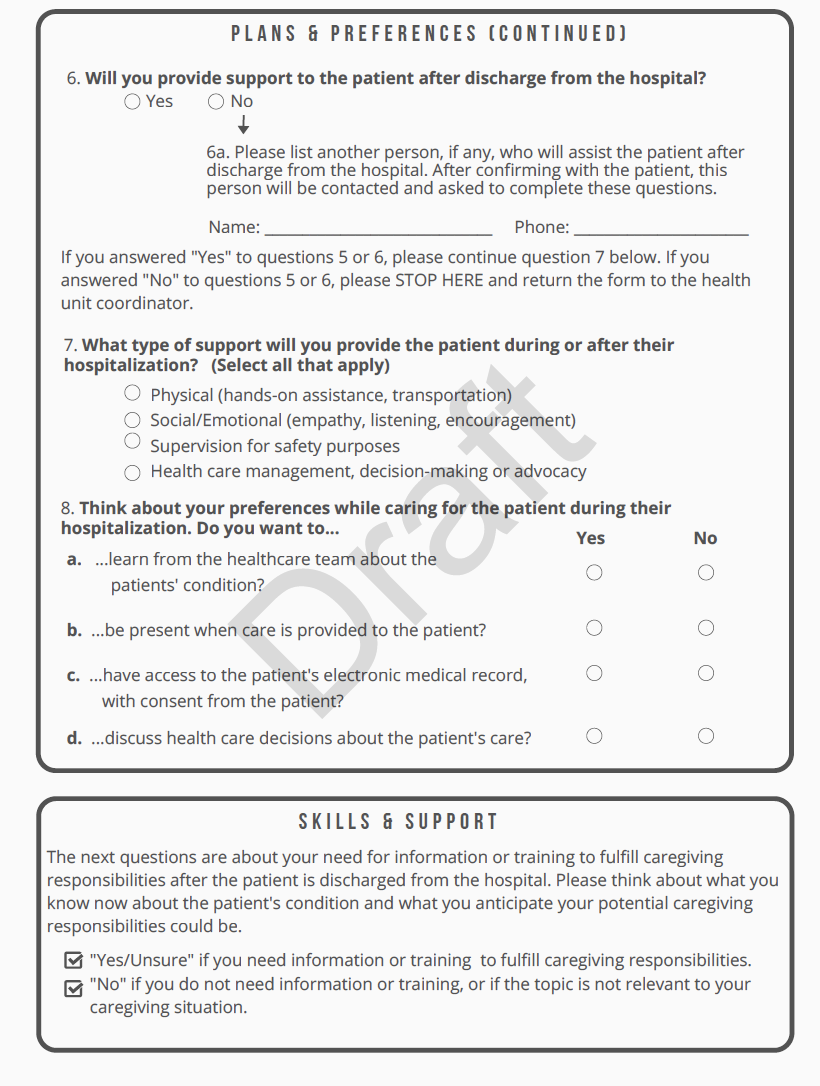
**

**
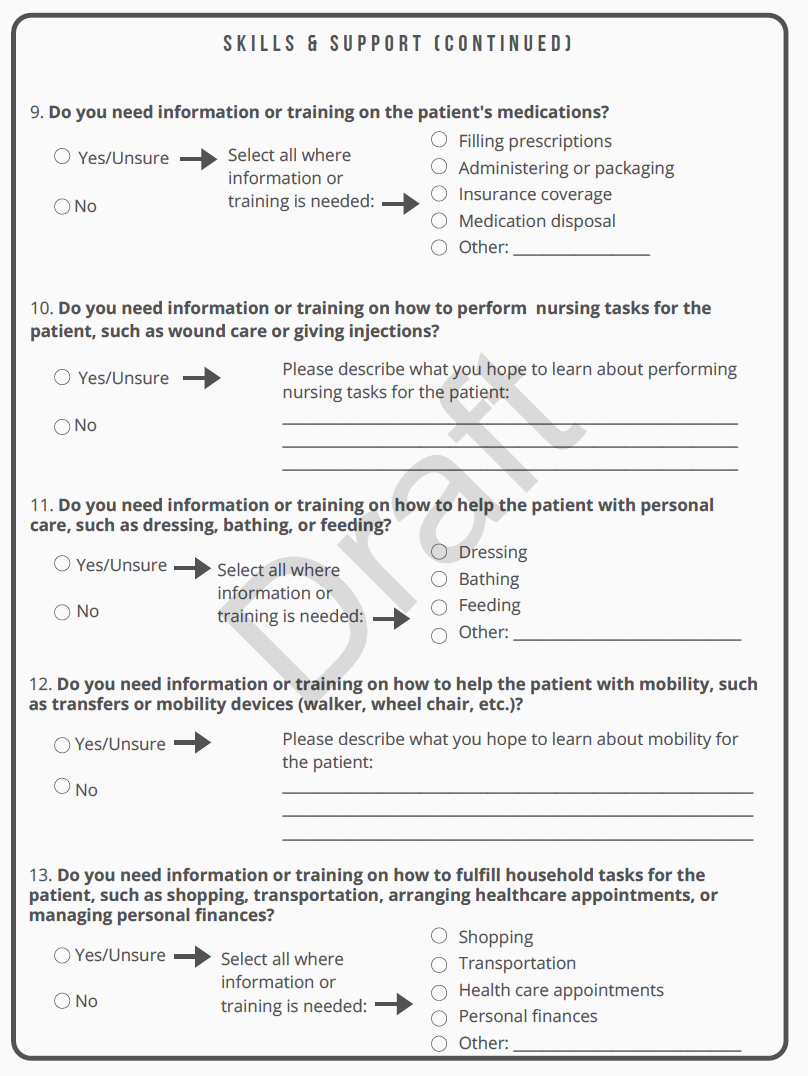
**

**
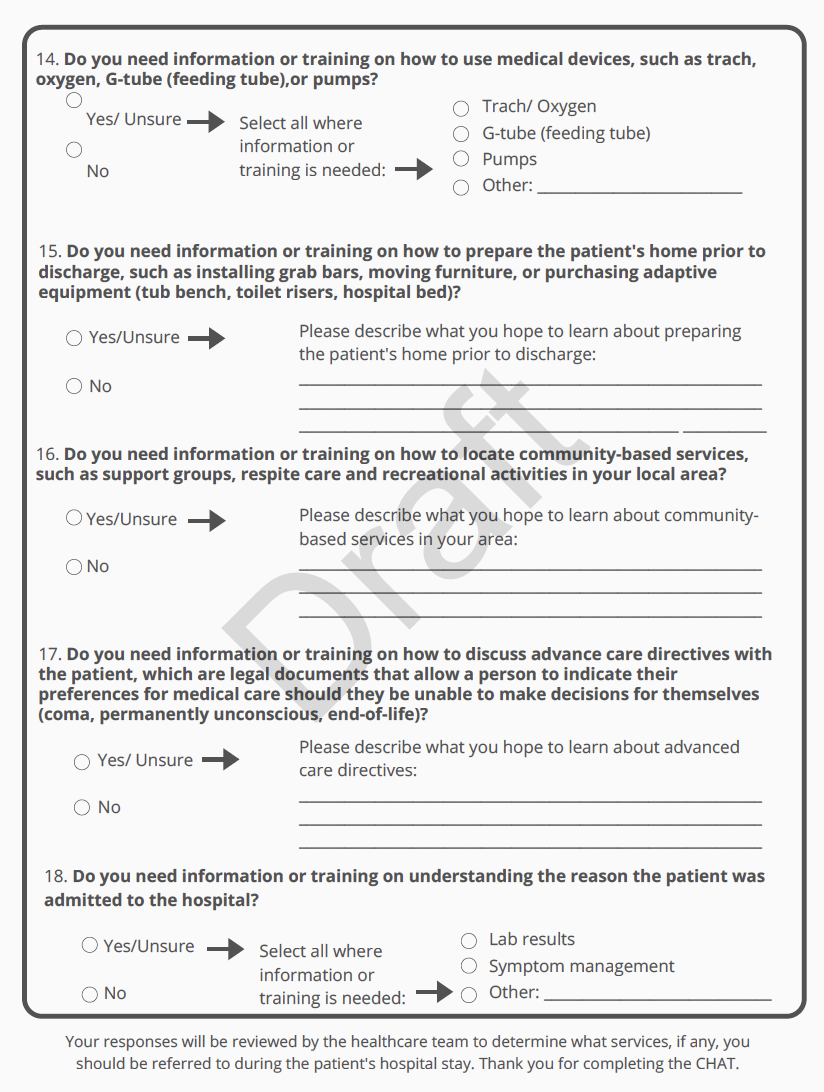
**

**
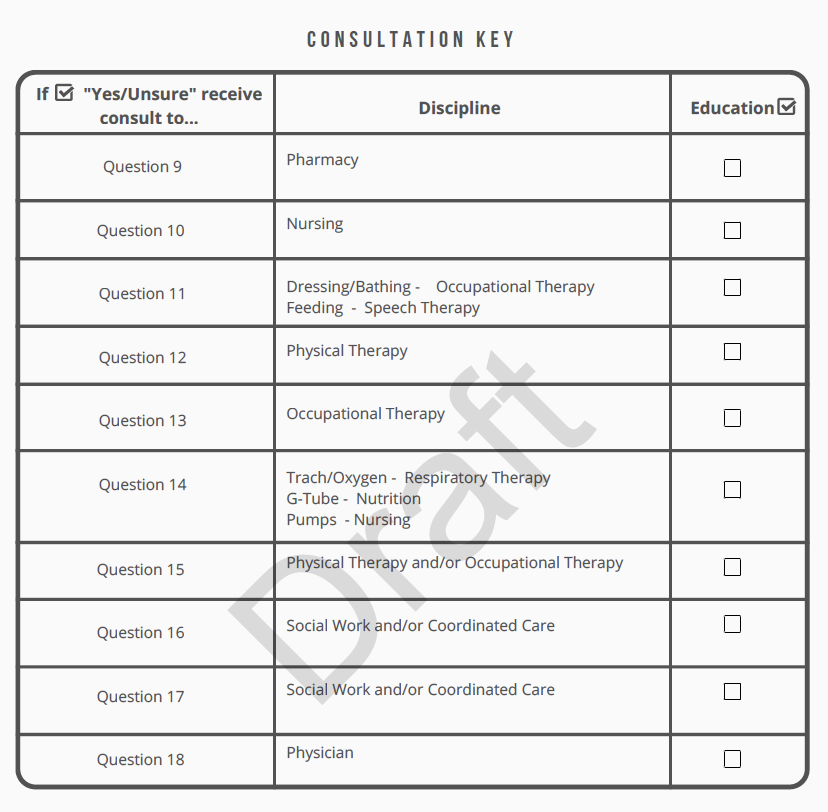
**

**Section 2:** Interview Guide

I’d like to start by learning your initial impressions of the tool.

1. Is there a strong need for this type of tool? Why or why not?
2. What are some of the things you liked about the tool?
   1. Content/clarity of instructions?
3. Are there things you didn’t like about the tool?
   1. Are there any suggestions to improve the content of the tool?
4. Are there important topics or ideas about caregiving that were missing from the tool?
5. Given the needs you identified, do you feel that your loved one’s health care team adequately addressed them? Why or why not?
   1. What barriers do you think impacted why your needs went unaddressed?
   2. Do you have any solutions for improving the delivery of the tool and/or addressing your needs?

Now, I’d like to hear about how you think the tool would affect you as a care partner.

1. Did completing the tool over the phone with us change your feelings or thoughts as a care partner?
2. How do you think the tool could be helpful to you in the future?
